# HDL-Mediated Lipid Influx to Endothelial Cells Contributes to Regulating Intercellular Adhesion Molecule (ICAM)-1 Expression and eNOS Phosphorylation

**DOI:** 10.3390/ijms19113394

**Published:** 2018-10-30

**Authors:** Mónica Muñoz-Vega, Felipe Massó, Araceli Páez, Gilberto Vargas-Alarcón, Ramón Coral-Vázquez, Jaime Mas-Oliva, Elizabeth Carreón-Torres, Óscar Pérez-Méndez

**Affiliations:** 1 Molecular Biology Department, Instituto Nacional de Cardiología “Ignacio Chávez”, 14080 Mexico City, Mexico; moni.muvega@gmail.com (M.M.-V.); gilberto.vargas@cardiologia.org.mx (G.V.-A.); elizact73@gmail.com (E.C.-T.); 2 Physiology Departments, Instituto Nacional de Cardiología “Ignacio Chávez”, 14080 Mexico City, Mexico; felipe.masso@cardiologia.org.mx (F.M.); araceli.paez@cardiologia.org.mx (A.P.); 3 Graduate School and Research Division, Escuela Superior de Medicina, Instituto Politécnico Nacional, 11340 México City, Mexico; rmcoralv@gmail.com; 4 Sub-Directorate of Research and Education, Centro Médico Nacional “20 de Noviembre”, Instituto de Seguridad y Servicios Sociales de los Trabajadores del Estado, 03100 México City, Mexico; 5 Instituto de Fisiología Celular, Universidad Nacional Autónoma de México, 04510 Mexico City, Mexico; jmas@ifc.unam.mx

**Keywords:** HMEC-1, inflammation, vascular cell adhesion molecule-1, SR-BI, HDL sphingomyelin, HDL cholesterol, atherosclerosis, reverse cholesterol transport, endothelial function

## Abstract

Reverse cholesterol transport (RCT) is considered as the most important antiatherogenic role of high-density lipoproteins (HDL), but interventions based on RCT have failed to reduce the risk of coronary heart disease. In contrast to RCT, important evidence suggests that HDL deliver lipids to peripheral cells. Therefore, in this paper, we investigated whether HDL could improve endothelial function by delivering lipids to the cells. Internalization kinetics using cholesterol and apolipoprotein (apo) AI fluorescent double-labeled reconstituted HDL (rHDL), and human dermal microvascular endothelial cells-1 (HMEC-1) showed a fast cholesterol influx (10 min) and a slower HDL protein internalization as determined by confocal microscopy and flow cytometry. Sphingomyelin kinetics overlapped that of apo AI, indicating that only cholesterol became dissociated from rHDL during internalization. rHDL apo AI internalization was scavenger receptor class B type I (SR-BI)-dependent, whereas HDL cholesterol influx was independent of SR-BI and was not completely inhibited by the presence of low-density lipoproteins (LDL). HDL sphingomyelin was fundamental for intercellular adhesion molecule-1 (ICAM-1) downregulation in HMEC-1. However, vascular cell adhesion protein-1 (VCAM-1) was not inhibited by rHDL, suggesting that components such as apolipoproteins other than apo AI participate in HDL’s regulation of this adhesion molecule. rHDL also induced endothelial nitric oxide synthase eNOS S1177 phosphorylation in HMEC-1 but only when the particle contained sphingomyelin. In conclusion, the internalization of HDL implies the dissociation of lipoprotein components and a SR-BI-independent fast delivery of cholesterol to endothelial cells. HDL internalization had functional implications that were mainly dependent on sphingomyelin. These results suggest a new role of HDL as lipid vectors to the cells, which could be congruent with the antiatherogenic properties of these lipoproteins.

## 1. Introduction

Reverse cholesterol transport (RCT) has been accepted as the most important activity of high-density lipoproteins (HDL) in atherosclerosis protection [1]. However, RCT only partially explains the beneficial activities of HDL on endothelium [1,2]. HDL promote nitric oxide (NO) synthesis in endothelial cells through interaction with scavenger receptor class B type I (SR-BI), which has become accepted as a canonical HDL receptor [3]. HDL-induced synthesis of NO, in turn, seems to contribute to the inhibition of the vascular cell adhesion protein-1 (VCAM-1) and the intercellular adhesion molecule-1 (ICAM-1) [4]. In keeping with this line of evidence, previous studies have reported HDL internalization by endothelial cells. Some authors have interpreted this process as only the transcytosis of HDL holoparticles through the endothelial barrier [3,5,6]. However, during such internalization, early and selective cholesterol influx occurs in the membranes and phospholipid bilayers [5,7], and it remains undetermined whether cholesterol influx is relevant for the inhibition of VCAM-1 and ICAM-1 expression and NO synthesis in the endothelial cells, as previously described [4,8].

There is some important evidence that suggests the role of HDL as a cholesterol vector from the liver to the tissues: (1) Most of the cholesterol mass in mammals is synthesized in the liver, and its distribution in the peripheral organs must be achieved by a lipoprotein of hepatic synthesis, i.e., HDL [9]. (2) The HDL receptor SR-BI has been proposed as a cholesterol sensor [10] and is expressed in almost all tissues [3]. (3) HDL becomes smaller after interaction with SR-BI, suggesting lipid uptake by the cells [11]. (4) Genetically compromised SR-BI function is associated with high HDL cholesterol plasma levels—instead of low plasma levels as expected—if the protein promotes cholesterol efflux [12]. Therefore, we hypothesized that HDL deliver cholesterol to the cells via SR-BI and that such lipid influx is of physiological relevance for endothelial function. Moreover, HDL sphingomyelin seems to play an important role in HDL functionality [13,14], most likely as the precursor of sphingosine 1-phosphate (S1P) [15] or by preserving the structure of lipid rafts in the cellular membrane [16]. Therefore, if HDL deliver cholesterol to the peripheral cells, these lipoproteins may also simultaneously be a vector for sphingomyelin. Such lipid influx to the cells may be related to the effect of HDL on endothelium function, i.e., the activation of endothelial nitric oxide synthase (eNOS) and the expression of adhesion molecules [2].

In this study, we looked for the cholesterol and sphingomyelin influx to endothelial cells mediated by HDL and whether this influx contributed to regulating the expression of ICAM-1 and VCAM-1 as well as the phosphorylation of eNOS in human dermal microvascular endothelial cells-1 (HMEC-1)-cultured cells. This study presents the first approach to exploring HDL-mediated lipid influx as a conceivable antiatherogenic function of these lipoproteins.

## 2. Results

### 2.1. Internalization of HDL Lipids and HDL Protein

**Figure 1:**
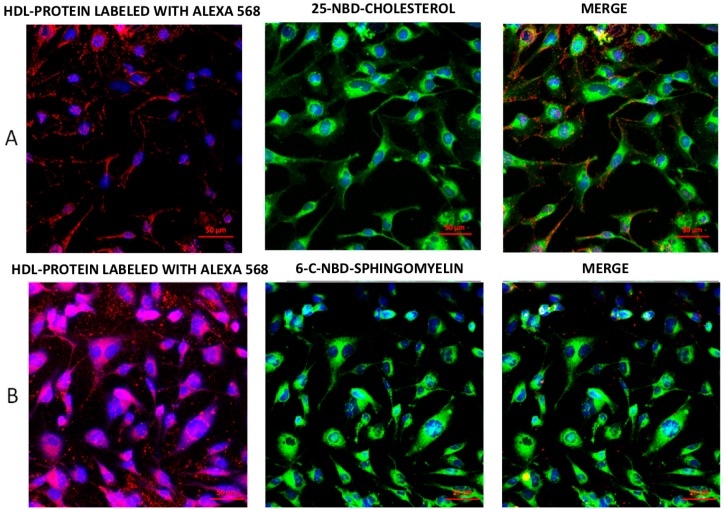
Representative confocal images of lipids and high-density lipoprotein (HDL) protein internalization in HMEC-1 after 20 min incubation with fluorescent double-labeled reconstituted HDL (rHDL). (**A**) Cholesterol and apo AI double-labeled rHDL showed that the cellular location of protein stained with Alexa 568 (red) followed a different distribution when compared with 25-NBD-cholesterol (green). (**B**) Incubation of human dermal microvascular endothelial cells-1 (HMEC-1) with rHDL containing C-6-NBD-sphingomyelin and HDL protein labeled with Alexa 568 fluorescent tracers. Both sphingomyelin and protein colocalized within the cells. Nuclei were labeled with 4′,6-diamidino-2-phenylindole (DAPI) (blue). Scale bars represent 50 μm.

We performed internalization assays using rHDL double-labeled as either protein and cholesterol or protein and sphingomyelin. Confocal images of representative experiments are shown in Figure 1A. The HDL protein labeled with Alexa 568 was internalized to the cytoplasm. Lipid components, represented by fluorescent cholesterol or sphingomyelin, were also internalized. The distribution of HDL cholesterol in comparison with HDL protein was different (Figure 1A); this observation was confirmed by the poor colocalization coefficient (*r* = 0.006). In contrast, the HDL sphingomyelin and HDL protein colocalized within the cell (*r* = 0.998) (Figure 1B). The single-labeled rHDL demonstrated similar distribution patterns to any of the lipoprotein components—protein, cholesterol, or sphingomyelin (Figure S1).

### 2.2. Kinetics of HDL Lipids Influx

**Figure 2:**
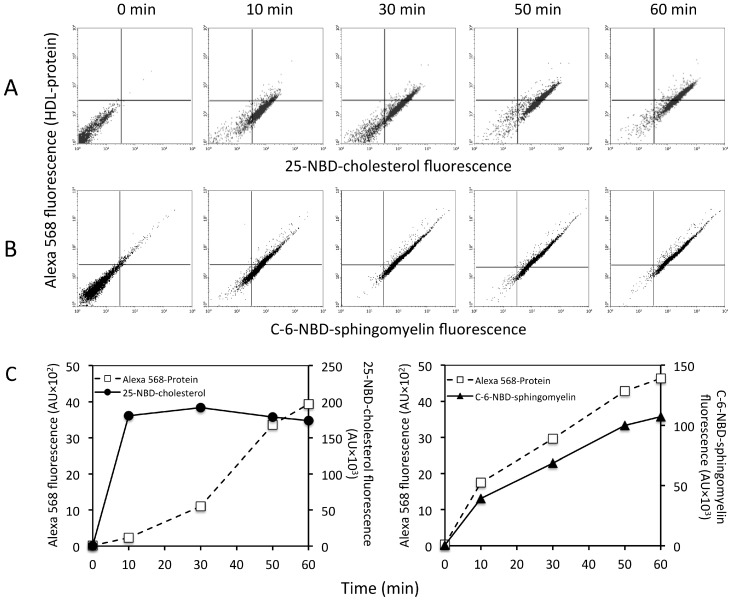
Kinetics of internalization assays performed by flow cytometry using double-labeled rHDL. HMEC-1 was incubated from 10 to 60 min with rHDL containing either (**A**) 25-NBD-cholesterol and HDL protein labeled with Alexa 568 or (**B**) C-6-NBD-sphingomyelin and HDL protein labeled with Alexa 568 fluorescent tracers. Cholesterol was rapidly associated with the cells from 10-min incubation with rHDL (right lower quadrants in the dot plots of row **A**), whereas protein began to be incorporated to HMEC-1 after 30 min of incubation (right upper quadrants). In contrast, both sphingomyelin and protein fluorescent signals were found associated with cells simultaneously (right upper quadrants in the dot plots of row (**B**). (**C**) The 60-min internalization kinetics of the 25-NBD-cholesterol and HDL protein labeled with Alexa 568 (Alexa 568 Protein) (**left**) and C-6-NBD-sphingomyelin and apo AI-Alexa 568 (**right**). Results are the mean of three independent experiments for each tested time.

Double-labeled rHDL preparations were used to assess the internalization kinetics along 60 min of each HDL component by flow cytometry in three independent experiments (Figure 2). The dot plot shows cells labeled early (10 min) with only 25-NBD-cholesterol (Figure 2A, right lower quadrants), whereas the Alexa 568-labeled HDL protein within the cells increased mainly after 30 min of incubation (Figure 2A, right upper quadrants). In contrast, the kinetics of HDL sphingomyelin internalization was different to that of cholesterol (Figure 2B). Double-labeled cell populations were the most abundant along the time of incubation (upper right quadrants in the plots), indicating that the fluorescence of HDL sphingomyelin increased concomitantly to that of HDL protein within the cells. The complete internalization kinetics is represented in Figure 2C. As expected, the HDL cholesterol followed different kinetics of internalization than the HDL protein, whereas the HDL sphingomyelin had a similar behavior to the latter.

### 2.3. HDL/LDL Cholesterol Competition Assays

**Figure 3:**
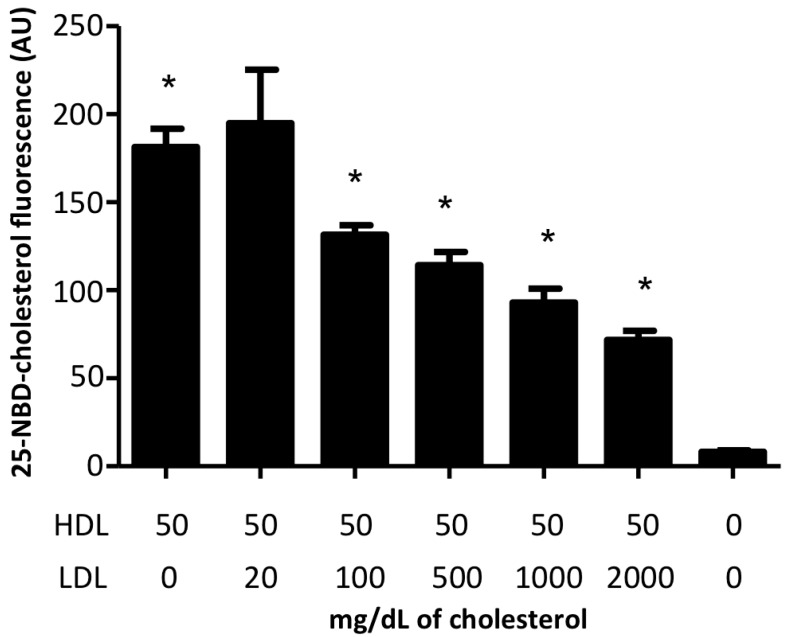
Competition assays between HDL cholesterol and low-density (LDL) cholesterol internalization in HMEC-1. Cultured cells were coincubated with a constant rHDL cholesterol concentration (50 mg/dL) and increasing concentrations (20 to 2000 mg/dL) of cholesterol associated with LDL isolated from human plasma. rHDL were labeled with 25-NBD-cholesterol, and internalization was monitored by flow cytometry. Data correspond to mean ± SD of three independent experiments. * *p* < 0.05 vs. HDL 50 mg/dL and no LDL.

In order to gain more insight into the relevance of cholesterol delivery to the cell by HDL vis-à-vis LDL, we performed competition assays using HDL labeled with 25-NBD-cholesterol at a fixed concentration (50 mg/dL of cholesterol) and increasing concentrations of LDL cholesterol in a range from 50 to 2000 mg/dL. Our results showed a dose-dependent decrease in HDL cholesterol internalization with increasing doses of LDL cholesterol. However, even when at 2000 mg/dL of LDL cholesterol (about 20 times the physiological concentration), cells continued internalizing cholesterol from HDL (Figure 3).

### 2.4. Contribution of SR-BI to HDL Internalization

**Figure 4:**
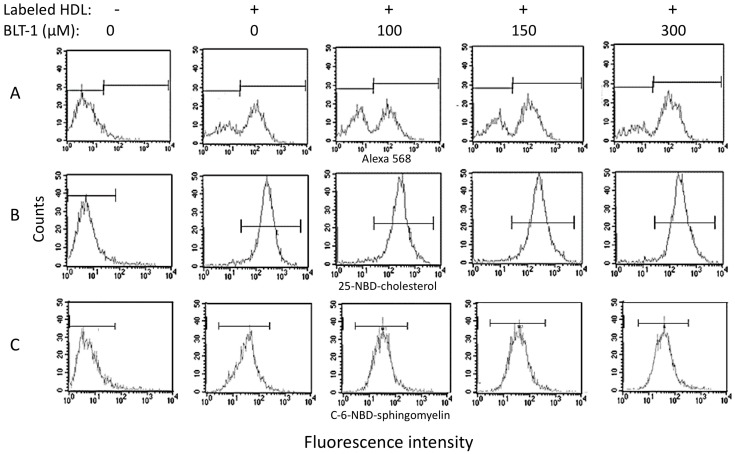
Contribution of scavenger receptor class B type I (SR-BI) to the uptake of different components of rHDL by HMEC-1 as determined by flow cytometry. Cells were incubated with single-labeled rHDL containing (**A**) Alexa-488-labeled apo AI, (**B**) 25-NBD-cholesterol, (**C**) C-6-NBD-sphingomyelin; different concentrations of block lipid transport-1 (BLT-1) are indicated at the top of the figure. Representative graphs of the number of events (counts, vertical axis) vs. fluorescence intensity (horizontal axis) are shown. Horizontal bars indicate the regions corresponding to cells that incorporated the fluorescent tracer (right bars) and nonlabeled cells (left bars). For C-6-sphingomyelin, labeled and nonlabeled cells overlapped in the experiments that included BLT-1.

As SR-BI is one of the few accepted HDL receptors, we looked for the contribution of this protein to the internalization of HDL to endothelial cells. These experiments were performed with three different concentrations (100, 150, and 300 μM) of the irreversible SR-BI-inhibitor, block lipid transport-1 (BLT-1), and apo AI-Alexa 568 or 25-NBD-cholesterol or C-6-NBD-sphingomyelin single-labeled HDL (Figure 4). The fluorescence of any of the three components was found within HMEC-1 cells after 30 min of incubation (peaks displaced to the right in Figure 4A–C). At 100 μM of BLT-1, we observed a decrease in the fluorescence corresponding to the labeled protein concomitantly with an increase in the peak of nonlabeled cells (Figure 4A, third panel). This result indicated that 100 μM of BLT-1 induced a partial inhibition of HDL protein internalization. Paradoxically, at higher concentrations of BLT-1, the inhibition of protein internalization did not increase as expected; instead, HDL protein internalization gradually rose in a BLT-1 dose-dependent manner (Figure 4A, fourth and third panels). In contrast to the HDL protein, the whole HMEC-1 cell internalized the 25-NBD-cholesterol, which was not affected by any of the three tested concentrations of BLT-1 (Figure 4B). Concerning sphingomyelin, the coincubation of cells with any of the three concentrations of BLT-1 resulted in wider peaks of labeled cells than those observed in the absence of the SR-BI inhibitor (Figure 4C). These wider peaks are indicative of a more heterogeneous population of cells in terms of the number of internalized fluorescent markers.

### 2.5. Inhibition of Adhesion Molecules by rHDL

**Figure 5:**
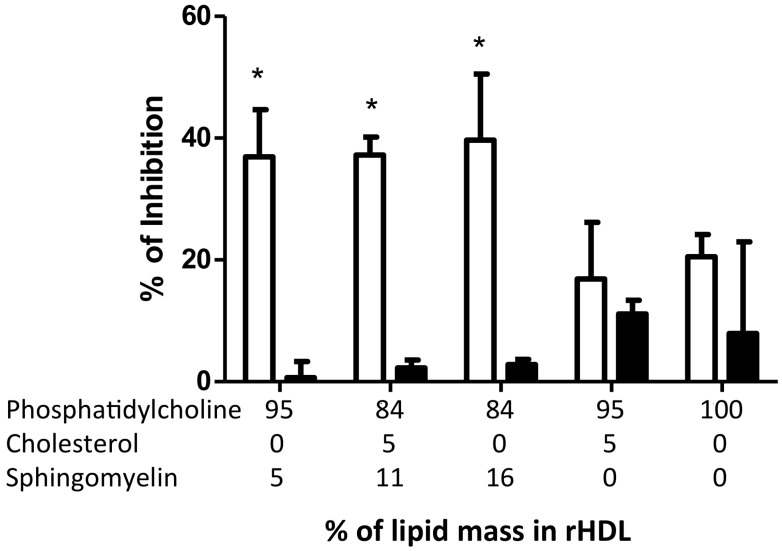
Inhibition of intercellular adhesion molecule-1 (ICAM-1) levels by rHDL after TNF-α stimulation. HMEC-1 was incubated for 4 h with rHDL containing different lipid proportions as indicated in the horizontal axis before TNF-α stimulation. Results are represented as the percentage of inhibition, considering the levels of ICAM-1 induced by TNF-α without rHDL as 100% of expression, then subtracting the ICAM-1 expression levels after rHDL incubation. Results were obtained using the mean of three independent experiments. * *p* < 0.05 vs. 100 PC/Apo AI.

To explore the potential functional role of HDL internalization, we analyzed the cell membrane expression of tumor necrosis factor (TNF)-α-induced ICAM-1 and VCAM-1 in HMEC-1 coincubated with rHDL containing different lipid compositions (Figure 5). Interestingly, rHDL containing sphingomyelin significantly inhibited ICAM-1 expression (−38%) in a non-dose-dependent manner (Figure 5). In agreement, rHDL lacking sphingomyelin had a poor effect on ICAM-1 expression (about 15% inhibition, *p* > 0.05), independent of the cholesterol content (Figure 5). On the other hand, VCAM-1 was not significantly inhibited by rHDL in these experiments.

### 2.6. eNOS Phosphorylation in the Presence of rHDL

**Figure 6:**
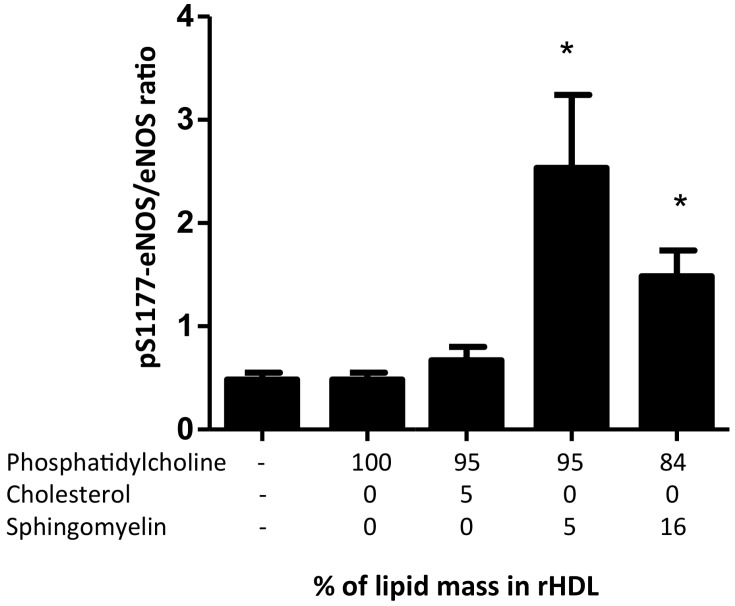
Phosphorylation of eNOS at S1177 after incubation with rHDL reconstituted with different lipid content (indicated in the horizontal axis). HMEC-1 was incubated for 20 min with rHDL containing different lipid proportions. Total eNOS and S1177-phosphorylated eNOS were determined by confocal microscopy using secondary antibodies labeled with Alexa Fluor 405 (blue) and fluorescein isothiocyanate (FITC, green), respectively. Bars represent the ratio of the fluorescence determined for S1177-phosphorylated eNOS (pS1177-eNOS) and the fluorescence of total eNOS detected. Fluorescence was adjusted by cell area and background and expressed as mean ± SD of three independent experiments. * *p* < 0.005 vs. control.

We further focused on the role of HDL in inducing the activation of eNOS, particularly on the phosphorylation of the residue S1177 (pS1177-eNOS). Microscopic analysis of HMEC-1 incubated with rHDL specifically stained for eNOS and pS1177-eNOS are shown in Figure S2. The densitometric analysis of the images demonstrated that the p1177-eNOS to eNOS ratio was dependent on the presence of sphingomyelin in rHDL (Figure 6); however, 5% of sphingomyelin content within rHDL had a better effect than rHDL containing 16% of the lipid. In contrast, rHDL structured with apo AI and phospholipids did not induce eNOS phosphorylation when compared with the control cells, independently of the presence of cholesterol (Figure 6).

## 3. Discussion

In the present study, we demonstrated that HDL protein and HDL lipids internalization in endothelial cells was a fast and sustained process with some physiological implications on cell function. This work was performed using HMEC-1, a cell line derived from dermal microvasculature that has been previously described and validated for lipoprotein studies [17]. HDL holoparticle internalization has been previously reported in several cell types, including endothelial cells, mainly with HDL labeled in the protein moiety using radioisotopes or fluorescent molecules [3,5,6,18]. Nonetheless, several questions in the field still remain, particularly regarding the role of lipid influx from HDL to the membranes [5,7]. Here, we demonstrated the internalization of three components of HDL—protein, cholesterol, and sphingomyelin—using rHDL of diverse compositions. To our knowledge, this is the first report regarding HDL sphingomyelin internalization.

HDL protein was internalized in discrete granules as reported previously [5,18], whereas HDL cholesterol content was diffused in the cytoplasm, suggesting different destinations for the HDL components. The colocation coefficient for the cholesterol and protein of HDL was very low, suggesting a dissociation of the particle during the internalization process as recently suggested [7,19]. Such dissociation of the protein and cholesterol moieties of HDL was further supported by the different kinetics of the two HDL components determined by flow cytometry. Interestingly, our results revealed that endothelial cells, independent of protein internalization, could quickly assimilate cholesterol from HDL. This observation was in agreement with the transfer of HDL lipids to phospholipid bilayers [7].

SR-BI has been proposed as the membrane receptor that mediates the induction of NO synthesis and the inhibition of VCAM-1 and ICAM-1 [4], suggesting that this protein contributes to HDL internalization. Therefore, to determine the contribution of SR-BI to the internalization of the HDL components, we performed assays using the irreversible SR-BI-inhibitor BLT-1 [20]. BLT-1 partially inhibited HDL protein internalization but, paradoxically, this inhibitory effect gradually decreased at higher concentrations of BLT-1. It is likely that the control of HDL uptake became impaired when SR-BI was blocked [10]; this idea implies additional pathways for HDL internalization that are not affected by BLT-1. In this regard, the purinergic receptor P2Y13 contributes to HDL uptake [21], probably in the synergic interaction with the ectopic β-chain of cell surface F(0)F(1) ATPase [22,23]. More studies are required to understand the SR-BI, P2Y13, and β-chain of ATPase action in concert for the internalization of HDL components.

Conversely, the HDL cholesterol intake by endothelial cells was not affected by any concentration of BLT-1. The internalization of cholesterol, but not of protein, in the presence of BLT-1 suggests that the influx of cholesterol contained in the HDL was independent of the SR-BI transporter. This observation was consistent with a recent study indicating that HDL interaction with synthetic lipid bilayers leading to cholesterol transfer to the membrane may occur in the absence of HDL receptors [7]. This SR-BI-independent mechanism may play a key role in cholesterol delivery from HDL to the cell membrane. However, recent studies have proposed that SR-BI is a cholesterol sensor in the cell membrane [10]. It is likely that under the conditions of the study (1 h cholesterol “fasting”), there was an early uptake of cholesterol from HDL (within 10 to 30 min and up to 100 min of incubation [6]), which was compensated by a posterior efflux via SR-BI commonly observed 4 to 6 h after cholesterol load [24] in order to adjust the content of lipids in the membrane. Therefore, HDL delivered cholesterol to endothelial cells in culture in an SR-BI-independent manner, and a further lipid adjustment may be SR-BI-dependent. Cholesterol intake from HDL by cultured cells occurred even in the presence of LDL up to 20 times the physiological concentration of LDL; this result was unexpected because LDL have been considered as the most important donor of cholesterol for peripheral cells [25]. The fact that cells preferred cholesterol from HDL rather than from LDL is in agreement with earlier studies, which demonstrated that HDL inhibited LDL uptake by bovine endothelial cells [26,27]. This suggests that HDL cholesterol has an important role in endothelial cell function.

In contrast to cholesterol, sphingomyelin and protein had the same distribution within the cell, suggesting that both components were internalized together. The kinetics of sphingomyelin incorporation to the cells followed the same pattern as that of the protein, supporting the idea that this lipid remained associated with HDL protein during cell internalization. In this regard, earlier studies have revealed that the interaction between sphingomyelin is very stable [28], and these data were consistent with our findings. As the HDL sphingomyelin kinetics of internalization overlapped that of the HDL protein, it was expected that inhibition of SR-BI would result in a partial inhibition of the incorporation to the cells, gradually reverted by increasing doses of BLT-1. Unexpectedly, BLT-1 did not inhibit HDL sphingomyelin internalization as observed for the HDL protein. Certainly, the peaks of flow cytometry became wider with BLT-1, indicating a more heterogeneous population of cells in terms of the quantity of internalized fluorescent sphingomyelin. These data suggest a regulation role of sphingomyelin uptake from HDL by the endothelial cells, which warrants further studies.

To explore the physiological impact of the internalization of HDL components in HMEC-1, we determined the capacity of rHDL to inhibit ICAM-1 and VCAM-1 expression as well as induce eNOS phosphorylation in S1177. Our data demonstrated that sphingomyelin was essential for rHDL to inhibit ICAM-1 expression, but not VCAM-1 expression, regardless of its proportion within the lipoproteins. It should be emphasized that the physiological content of sphingomyelin in HDL is between 5 and 15% [29], similar to the rHDL sphingomyelin content in our experiments. There is some evidence indicating that ICAM-1 is associated with lipid-raft-containing sphingolipids [8,30,31], suggesting that sphingomyelin delivered to the endothelial cells contributes to ICAM-1 inhibition. This idea is consistent with previous studies that showed that native HDL, rHDL, or proteoliposomes containing sphingomyelin inhibited ICAM-1 [4,32]. However, in contrast with our results, Kimura et al. [4] reported that rHDL inhibited the expression of VCAM-1. This apparent inconsistency with our results may be explained by the protein content of rHDL; whereas Kimura et al. used the whole delipidated protein fraction—which certainly contained several proteins—to reconstitute their HDL, we only used purified Apo AI for rHDL. Therefore, it seems that the inhibition of VCAM-1 by rHDL is dependent on a protein component of HDL other that Apo AI and is independent of sphingomyelin content in these lipoproteins.

There is some evidence indicating that eNOS is associated with lipid-raft-containing sphingomyelin [8], suggesting that this lipid, delivered to the endothelial cells by HDL, contributes to the eNOS activated form. The vasodilation induced by HDL elicits the phosphorylation of eNOS in serine 1177 via the S1P receptor [33]. Our data showed that the physiological content of sphingomyelin present in rHDL corresponded to an increased phosphorylation of eNOS. Our data were in agreement with the eNOS increased activity in CHO cells expressing SR-BI and transfected with bovine eNOS [8]. Moreover, as sphingomyelin is the precursor of S1P, our results were also consistent with previous reports [34]. However, eNOS phosphorylation was not sphingomyelin dose-dependent; in fact, the phosphorylation tended to decrease at a higher sphingomyelin content of rHDL. In this context, decreases in HDL sphingomyelin plasma concentrations have been associated with blood pressure improvement [14]. Therefore, all these results suggest that the beneficial effects of HDL-sphingomyelin in terms of vasodilation occur only within a tight interval of the lipid content in the HDL structure.

Although it is known that NBD-labeled lipids are more bulky and hydrophobic than the nonlabeled molecules [34], several functional studies have shown that the fluorescent probes used in this study somehow overlaps the behavior of the nonlabeled lipids [5,17].

Taken together, our results suggest a new role of HDL as lipid vectors to the cells. HDL lipid influx is congruent with the fast cholesterol segregation from HDL as recently described in Reference [19]. The role of HDL as lipid vectors is also consistent with the so-called “dysfunctional HDL” [35], which may internalize harmful molecules to the cells, e.g., oxidized lipids, miRNAs, and high content of triglycerides among others. The participation of HDL as lipid vectors in the atherosclerosis process merits further exploration.

In summary, we have demonstrated that the internalization of HDL to endothelial cells in culture implies the dissociation of the lipoprotein components, which leads to a fast delivery of cholesterol to the cells. Such cholesterol internalization is independent of both SR-BI and LDL, even at very high concentrations. HDL internalization has functional implications, including the regulation of ICAM-1 expression and eNOS phosphorylation, which are mainly dependent on sphingomyelin. By driving lipids to the cells, HDL may regulate endothelial function, and this mechanism is congruent with the antiatherogenic role of these lipoproteins.

## 4. Materials and Methods

### 4.1. Reagents

Medium MCDB-131 with phenol red was purchased from Gibco, Thermo Scientific, Grand Island NY. Fetal calf serum was from Hyclone, GE Healthcare, Little Chalfont, Buckinghamshire, UK. Antibiotic mix was from Gibco, Thermo Scientific, Carlsbad, CA, USA. L-glutamine, porcine hydrocortisone, and endothelial cell growth supplement were purchased from Sigma-Aldrich, St. Louis, MO, USA. The molecular probes labeling kit Alexa 488 or Alexa 568 were from Life Technologies, Eugene, OR, USA.

Fluorescent lipids, C-6-NBD-sphingomyelin (N-[6-[(7-nitro-2-1,3-benzoxadiazol-4-yl)amino]hexanoyl]-sphingosine-1-phosphocholine), and 25-NBD-cholesterol (25-[N-[(7-nitro-2-1,3-benzoxadiazol-4-yl)methyl]amino]-27-norcholesterol) were purchased from Avanti, Polar Lipids, Alabaster, AL, USA.

Cholesterol, sphingomyelin, and phosphatidylcholine, both from chicken egg yolk and Apo AI purified from human plasma, were purchased from Sigma-Aldrich, St. Louis, MO, USA. Cholesterol was determined with a commercial kit from Randox Laboratories Ltd. (County Antrim, UK), and the kit to determine phospholipids was from Wako Diagnostics, Wako Life Science, Inc., Mountain View, CA, USA.

TNF-α was purchased from Boehringer-Mannheim Biochemistry, Mexico City, Mexico. Antibodies anti-ICAM-1 labeled with fluorescein isothiocyanate (FITC) and anti-VCAM-1 associated with phycoerythrin (PE) were from BioLegend, San Diego, CA, USA. The antibody against eNOS and pS1177-eNOS was from Abcam (Cambridge, UK), and secondary antibodies anti-rabbit IgG labeled with FITC and anti-mouse IgG labeled with Alexa Fluor 405 were from Santa Cruz Biotechnology, Dallas, TX, USA.

### 4.2. Cell Culture

HMEC-1 (ATCC CRL-3243) was cultured at 37 °C with 7% pCO_2_ in a humidified atmosphere using phenol red containing MCDB-131 medium supplemented with 15% fetal calf serum (FCS), penicillin–streptomycin mixture 100 U/mL, L-glutamine 10 Mm, porcine hydrocortisone 1 μg/mL, and 20 μg/mL endothelial cell growth supplement [17,36].

### 4.3. Lipoprotein Isolation and Labeling

Isolation of native total LDL and total HDL were performed by sequential ultracentrifugation (LDL d = 1.019–1.063 g/mL, HDL d = 1.063–1.21 g/mL) as previously described in References [14,37] using plasma from healthy volunteers without dyslipidemias (cholesterol < 200 mg/dL, triglycerides < 150 mg/dL), metabolic abnormalities such as thyroid, liver, or renal dysfunction, and no family history of cardiovascular disease or diabetes. Lipoproteins were dialyzed with PBS-saline solution, and protein content was quantified by Lowry’s method. For the internalization assays, HDL were labeled in their protein portion using the Alexa 488 or Alexa 568 protein labeling kit following the manufacturer’s instructions with slight modifications as previously reported [17,22].

### 4.4. Synthesis of Reconstituted HDL (rHDL)

rHDL were prepared using the sodium cholate dialysis method previously described [38,39]. For the fluorescence assays, we prepared rHDL with phosphatidylcholine (PC), Apo AI, 11% C-6-NBD-sphingomyelin, and either 5% cholesterol or 2.5% 25-NBD-cholesterol/2.5% of cholesterol. These were the percentages of total lipid mass. All preparations included 0.5 mg of Apo AI.

To explore the effect of HDL composition on the expression of adhesion molecules and eNOS activation, we used rHDL prepared with variable cholesterol (CH) and sphingomyelin (SM) compositions: 100% PC/ApoAI, 95% PC/5% SM/ApoAI, 84% PC/11% SM/5% CH/ApoAI, 84% PC/16% SM/Apo AI, and 95% PC/5% CH/ApoAI. Proportions represent the percentage of total lipid mass.

Determinations of protein were performed with Lowry’s method, and phospholipid and cholesterol content were determined with commercial kits. SM was determined as previously reported by us [14].

### 4.5. Inhibition of Adhesion Molecules

HMEC-1 was plated using a density of 100,000 cell/mL and cultured overnight at 37 °C in a humidified atmosphere with 7% pCO_2_. Medium was replaced with MCDB-131 supplemented with lipoprotein-poor FBS and containing rHDL at a final concentration of 100 µg/mL of protein. After 4 h of incubation, cells were stimulated with 15 ng/mL TNF-α and incubated again for 5 h. Cells were fixed using 3.7% paraformaldehyde in PBS 10 mM, and recovered from the plate using collagenase II treatment, washed, and labeled with the antibodies anti-ICAM-1-FITC and anti VCAM-1-PE. Cells were washed and suspended in PBS 10 mM (1% bovine serum albumin) and 0.01% sodium azide buffer and analyzed by flow cytometry using a FACS Calibur equipment (Becton Dickinson, NJ, USA).

### 4.6. HDL Internalization Assay

HDL internalization assays were performed as previously described [17,23] with slight modifications for endothelial cells. Briefly, we incubated cells with MCDB-131 medium supplemented with 7% of lipoprotein-deficient FBS for 1 h. After this period, we slowly added rHDL or rHDL labeled in the protein moiety with Alexa Fluor 568 at a final protein concentration of 100 µg/mL and incubated for 20 min at 37 °C. Then, to minimize the unspecific unions between rHDL/HDL and the plasmatic membrane, we incubated cells at 4 °C for 1 h. Cells were fixed with 3.7% paraformaldehyde and then analyzed. Images were obtained using an LSM-700 Carl Zeiss confocal microscope (Carl Zeiss, Baden-Württemberg, Germany); scale bars were included using the ZEN Carl Zeiss software. Confocal analysis demonstrated that there was no superficial labeling outside the cells. Cells were alternately recovered from plates using type II collagenase (0.4 mg/mL), and then cell suspension was washed. We analyzed 5000 gated events by flow cytometry, discarding the cell debris and cell associations. Cell morphology, size, and granularity were assessed, and they were normal in all cases.

To analyze the SR-BI contribution to lipid influx in HMEC-1, we used block lipid transport-1 (BLT-1) at final concentrations of 100, 150, and 300 μM before performing the internalization assay.

### 4.7. Kinetics of HDL Lipids Influx

Kinetic assays were performed at 10, 30, 50, and 60 min of incubation time using 25-NBD-cholesterol and Alexa Fluor 568 double-labeled rHDL. After the incubation time, cells were recovered and analyzed by flow cytometry. We took 5000 gated events into account, discarding cell debris and cell associations. Cell morphology was assessed during the entire protocol, and cell size and granularity were verified.

### 4.8. HDL/LDL Cholesterol Competition Assays

We performed an internalization assay with rHDL containing 25-NBD-cholesterol as described above, at a constant concentration of cholesterol (50 mg/dL) and LDL isolated from the plasma of healthy volunteers at increasing concentrations of cholesterol, from 50 mg/dL to 2000 mg/dL. The amount of internalized cholesterol by the cells was estimated by flow cytometry, taking into account 5000 gated events as described above.

### 4.9. S1177-Phosphorylated eNOS Quantitation

Cells were incubated with rHDL containing different lipid compositions following the protocol described for the internalization assay using HDL isolated from human plasma as the control. eNOS and S1177-phosphorylated eNOS were determined by confocal microscopy using primary monoclonal antibodies and secondary antibodies labeled with Alexa Fluor 405 and FITC, respectively. Fluorescence quantification of confocal microscopy images was performed with ImageJ software (Bethesda, MD, USA), and values were adjusted by cell area and background; ZEN Carl Zeiss software (Baden-Württemberg, Germany) was used to indicate the scale bars.

### 4.10. Statistical Analysis

Statistical analysis was performed using Graph Pad Prism 5.0 software (La Jolla, CA, USA). Kruskall-Wallis nonparametric analyses were used for comparisons between groups, and results were expressed as the median when the data did not behave according to a standard distribution.

## Supplementary Materials

The following are available online at http://www.mdpi.com/1422-0067/19/11/3394/s1, Figure S1. Confocal images showing the internalization of single-labeled HDL components by HMEC-1. (**A**) rHDL containing protein labeled with Alexa 488 (green). (**B**) rHDL including fluorescent 25-NBD-cholesterol (green). (**C**) Internalization of HDL sphingomyelin after incubating with rHDL prepared with fluorescent C-6-NBD-sphingomyelin (green). In all cases, the cell nuclei were stained with DAPI (blue). Scale bars represent 20 μm. Figure S2. Representative images analyzed by confocal microscopy corresponding to the phosphorylation of eNOS in S1177 after incubation with rHDL prepared with different lipid proportions. eNOS and S1177-eNOS were revealed by immunocytochemistry using primary monoclonal antibodies and secondary antibodies labeled with FITC (green). Total eNOS levels were identified by immunocytochemistry using secondary antibodies labeled with Alexa 405 (blue). Scale bars represent 20 μm.
