# Supplementary material for: HDL-Mediated Lipid Influx to Endothelial Cells Contributes to Regulating Intercellular Adhesion Molecule (ICAM)-1 Expression and eNOS Phosphorylation
Source: Int J Mol Sci. 2018 Oct 30;19(11):3394. doi: 10.3390/ijms19113394 (PMC6274843; doi:10.3390/ijms19113394)
Supplement: Supplementary file 1 [file ijms-19-03394-s001.docx]

**Supplementary Figures**


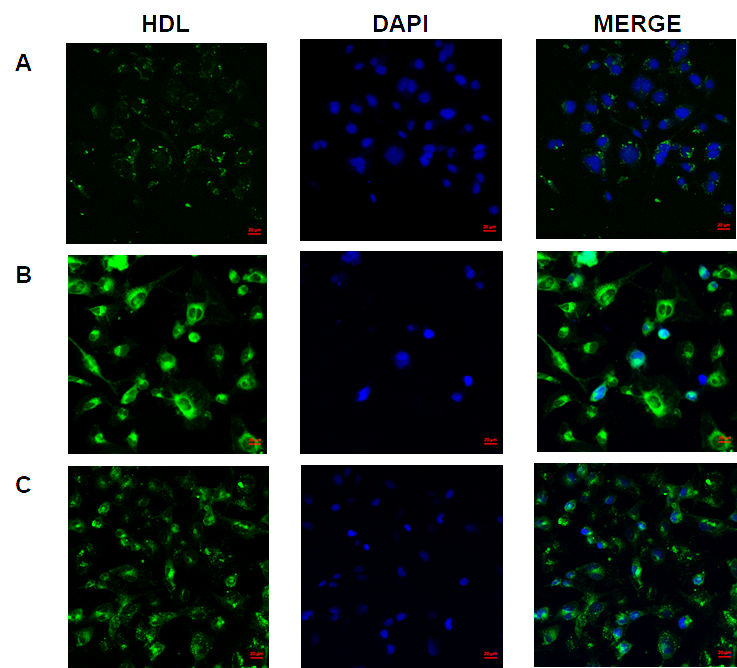


Figure S1. Confocal images showing the internalization of single-labeled HDL components by HMEC-1. A) rHDL containing protein labeled with Alexa 488 (green). B) rHDL including fluorescent 25-NBD-cholesterol (green). C) Internalization of HDL sphingomyelin after incubating with rHDL prepared with fluorescent C-6-NBD-sphingomyelin (green). In all cases cell nuclei were stained with DAPI (blue). Scale bars represent 20 μm

Figure S2. Representative images analyzed by confocal microscopy corresponding to the phosphorylation of eNOS in S1177 after incubation with rHDL prepared with different lipid proportions. eNOS and S1177-eNOS were reveled by immunohistochemistry using primary monoclonal antibodies and secondary antibodies labeled with FITC (green). Total eNOS levels were identified by immunohistochemistry using secondary antibodies labeled with Alexa 405 (blue). Scale bars represent 20 μm.
